# Macrophage-driven pathological ossification of the posterior longitudinal ligament: mechanistic insights and therapeutic opportunities

**DOI:** 10.1038/s41413-026-00576-8

**Published:** 2026-09-02

**Authors:** Huohuo Xue, Tsutomu Endo, Ken Kadoya, Norimasa Iwasaki, M. Alaa Terkawi

**Affiliations:** Department of Orthopedic Surgery, Faculty of Medicine and Graduate School of Medicine, Hokkaido University, Sapporo, Japan

**Keywords:** Metabolic syndrome, Fat metabolism, Bone

## Abstract

Ossification of the posterior longitudinal ligament (OPLL) is a chronic degenerative spinal condition in which bone gradually forms within the ligament, compressing the spinal cord and causing progressive neurological decline. While genetic predisposition and metabolic disturbances are well-recognized contributors, growing evidence points to macrophages as central players in shaping the tissue environment that drives pathological bone formation. Macrophages are remarkably plastic cells whose phenotypes and functions are continuously molded by cues from their local tissue environment. While pro-inflammatory macrophages sustain chronic low-grade inflammation through cytokine release and matrix remodeling, anti-inflammatory macrophages paradoxically amplify disease progression by driving fibrotic remodeling, excessive matrix accumulation, and aberrant vascularization, collectively fostering a hypoxic, ossification-prone niche within the ligament. Supporting this notion, similar macrophage-mediated mechanisms have been described in heterotopic ossification, though OPLL represents a distinct pathological process. This review synthesizes current evidence on how macrophages shape the OPLL microenvironment, draws mechanistic connections between macrophage activity and disease progression, and explores whether targeting these cells could open new therapeutic avenues.

## Introduction

Ossification of the posterior longitudinal ligament (OPLL) is a degenerative spinal disorder characterized by heterotopic bone formation within the posterior longitudinal ligament (PLL), leading to progressive spinal cord compression and neurological dysfunction. Early-stage OPLL is often asymptomatic; however, ossification lesions gradually enlarge with age, ultimately impairing mobility and quality of life. Epidemiological studies consistently demonstrate a markedly higher prevalence of OPLL in East Asian populations, suggesting a strong genetic contribution.^1^ Reported prevalence rates range from 1.9% to 4.3% in Japan, 1.1% to 1.7% in China, and 0.9% to 3.6% in Korea, compared with substantially lower rates of approximately 1.3% in Western populations.^1,2^

Accumulating evidence indicates that OPLL is a multifactorial disease arising from complex interactions among genetic, metabolic, mechanical, and inflammatory factors. Genome-wide association studies (GWAS) in Japanese cohorts have identified several susceptibility loci, including *RSPO2*, *SNX17*, and *SUPT3H*, underscoring the importance of genetic regulation of endochondral ossification pathways.^3,4^ Extending these genetic insights, a recent GWAS demonstrated that elevated body mass index is a causal determinant of diffuse spinal involvement in Japanese patients with thoracic OPLL.^5^ Supporting this, cohort studies have identified visceral fat obesity as a strong predictor of OPLL, particularly its diffuse form.^6,7^ East Asian populations are predisposed to visceral fat accumulation even at relatively lower body mass index levels and may exhibit reduced genetic resilience to metabolic syndrome,^8–11^ a susceptibility that likely contributes to the higher prevalence of OPLL in these populations compared with Western cohorts.^6,7^

Recent genomic analyses of thoracic OPLL have further highlighted the involvement of immune and myeloid-lineage cells in disease susceptibility. Polygenic risk has been shown to be significantly enriched in active enhancer regions of immune and hematopoietic cell populations, indicating that OPLL-associated variants act preferentially within these lineages in addition to mesenchymal and bone-related cells.^5^ Complementing this, integrative single-cell transcriptomic analyses in a heterotopic ossification model have shown that several OPLL susceptibility genes are expressed within immune cell clusters, including myeloid-lineage populations.^5^ Together, these findings support the concept that monocyte–macrophage lineages actively participate in shaping the inflammatory and osteogenic microenvironment that characterizes OPLL. Supporting this concept, clinical studies have linked OPLL to a constellation of metabolic disorders, including diabetes, obesity, visceral adiposity, dyslipidemia, and hepatic steatosis, all of which are characterized by chronic low-grade inflammation (LGI).^2,12–14^ Macrophages play a central role in LGI through their secretion of pro-inflammatory cytokines, including tumor necrosis factor-α (TNF-α), interleukin-1β (IL-1β), IL-6, IL-8, and monocyte chemoattractant protein-1, which collectively sustain chronic inflammatory signaling and shape a pro-osteogenic microenvironment.^15–18^

**Fig. 1:**
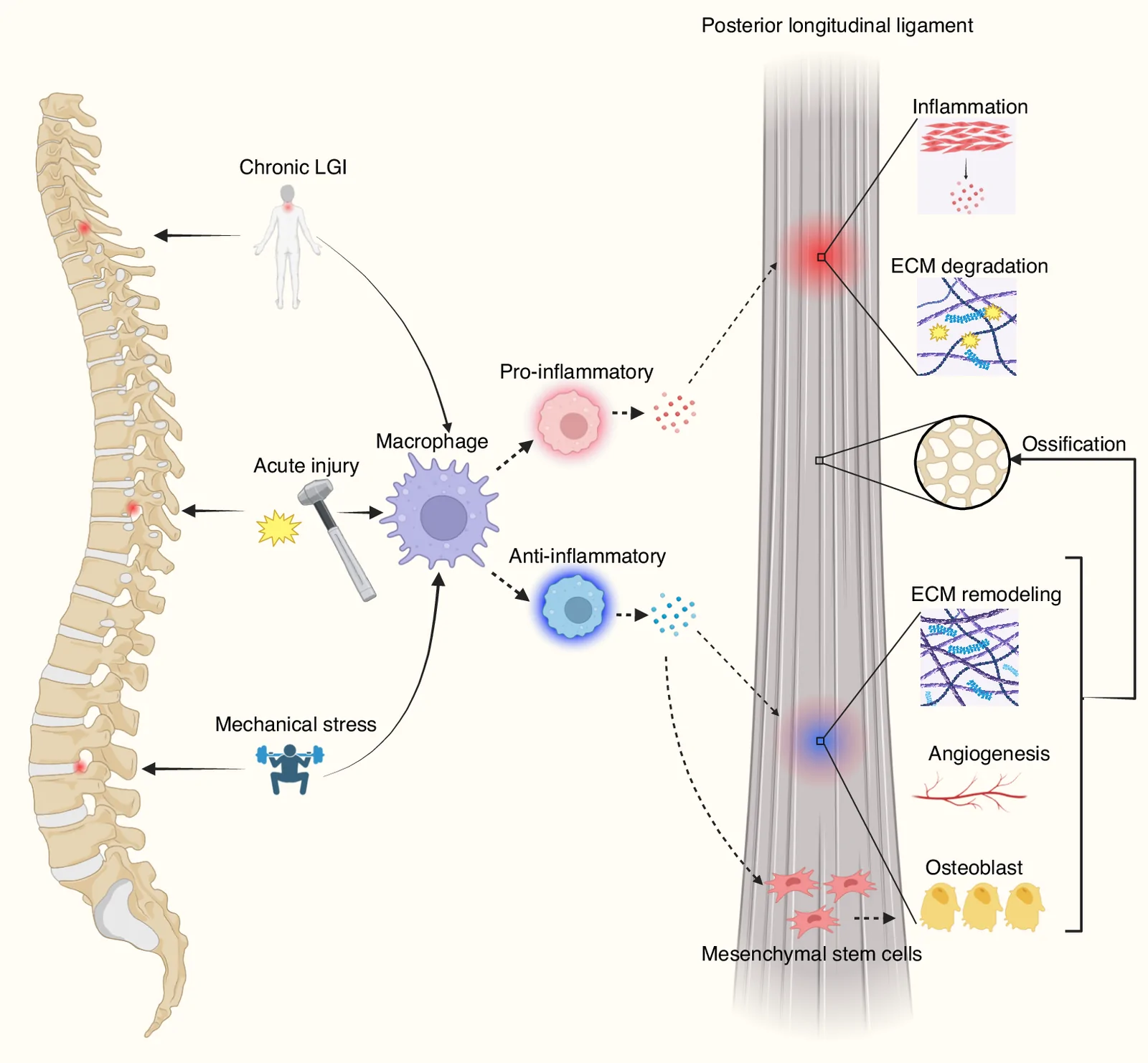
Schematic illustration of factor-mediated macrophage polarization in spinal ligament ossification. External stimuli, including mechanical stress, metabolic dysregulation, and chronic inflammation, trigger inflammatory responses within spinal ligament tissues, leading to the recruitment of circulating monocytes and their differentiation into tissue macrophages. Local microenvironmental cues subsequently drive polarization into functionally distinct phenotypes. Pro-inflammatory macrophages secrete cytokines such as tumor necrosis factor-α (TNF-α), interleukin-1β (IL-1β), and interleukin-6 (IL-6), which amplify inflammatory cascades, and release matrix metalloproteinases (MMPs) that degrade the damaged extracellular matrix. Anti-inflammatory macrophages, in contrast, produce interleukin-10 (IL-10) and transforming growth factor-β (TGF-β) to resolve inflammation and orchestrate extracellular matrix (ECM) remodeling. They also secrete growth factors, including bone morphogenetic protein-2 (BMP-2) and vascular endothelial growth factor (VEGF), which drive mesenchymal stem cell (MSC) differentiation toward an osteoblastic fate, together establishing a fibro-osteogenic microenvironment that supports ectopic bone formation

Mechanical stress represents another well-established risk factor in OPLL pathogenesis, acting through multiple PLL-resident cell populations. Although ligament fibroblasts are the predominant mechanosensitive cell type within the PLL, macrophages are also capable of responding to mechanical stimuli, with downstream effects on their function, morphology, and polarization state. Importantly, these mechanosensitive responses are directionally aligned with the osteogenic trajectory of PLL ossification, including the release of cytokines and tissue-remodeling enzymes, promotion of mesenchymal stem cell (MSC) differentiation toward an osteoblastic phenotype, and stimulation of angiogenesis.^19–21^ A growing body of evidence indicates that the macrophage secretome activates osteogenic signaling cascades such as wingless-type integration site (Wnt) and c-Jun N-terminal kinase, which have been linked to heterotopic ossification in conditions including vascular calcification, valvular sclerosis, and tendon calcification.^22,23^ These findings are reinforced by experimental models in which cyclic stretching of spinal ligaments activates osteogenic signaling cascades, particularly the transforming growth factor-β (TGF-β)/Smad and Wnt/β-catenin pathways.^19,20^ Consistent with these mechanisms, OPLL tissues show increased expression of osteogenic genes, including Runt-related transcription factor 2 (*RUNX2*), Osterix (*SP7*), Alkaline phosphatase (*ALPL*), and Osteocalcin (*BGLAP*), alongside upregulation of key signaling pathways such as bone morphogenetic protein (BMP)/TGF-β, mechanistic target of rapamycin (mTOR), and Wnt/β-catenin, all of which are subject to macrophage-mediated regulation.^19,20,24^ Together, these observations position macrophage-derived secreted factors as key regulators of bone metabolism and osteogenesis across diverse musculoskeletal disorders, including OPLL (Fig. 1).

Despite compelling evidence implicating macrophages as central mediators linking metabolic dysregulation, chronic inflammation, mechanical stress, and heterotopic ossification, their precise functional roles in OPLL remain incompletely defined. This review, therefore, focuses on the emerging roles of macrophages in OPLL pathogenesis, proposing an integrated framework in which macrophages serve as both key contributors to disease progression and as candidate therapeutic targets, warranting further investigation.

## Macrophage characteristics and their putative functions in OPLL pathogenesis

### Heterogeneity and function in tissues

Macrophages are versatile sentinels of the innate immune system that play essential roles in host defense, tissue homeostasis and development, and the pathogenesis of numerous diseases.^25,26^ They originate from yolk sac, fetal liver, and bone marrow progenitors and are widely distributed across tissues and organs, where they exhibit distinct tissue-specific characteristics shaped by their developmental origins.^27^ Their diverse functions rely on remarkable plasticity, which enables them to adapt to various tissue microenvironments by polarizing into distinct functional states in response to environmental cues, a dynamic process regulated by complex signaling pathways and transcriptional networks. Upon tissue injury, circulating monocytes are recruited to affected tissues, where they differentiate into subpopulations that functionally resemble resident macrophages and contribute to local immune responses.^28^

Although macrophages have traditionally been classified into classically activated (M1) and alternatively activated (M2) phenotypes, this binary framework is now widely recognized as an oversimplification, largely reflecting in vitro experimental conditions rather than the complexity of living tissues. In reality, macrophages exist across a broad continuum of transcriptional and functional states, continuously shaped by combinatorial signals from their microenvironment, including cytokines, metabolites, extracellular matrix (ECM) components, and mechanical forces.^29,30^ While exposure to lipopolysaccharide and interferon-γ (IFN-γ) drives macrophages toward a pro-inflammatory M1-like state, and IL-4 stimulation promotes an anti-inflammatory M2 phenotype, single-cell RNA sequencing and high-dimensional proteomics have made clear that tissue macrophages in vivo rarely conform to either pole. Instead, they adopt context-specific activation states that co-express genes associated with both classical and alternative activation, resisting simple classification. At the pro-inflammatory end of this spectrum, macrophages rewire their metabolism toward aerobic glycolysis and hypoxia-inducible factor-1α (HIF-1α) activation, a shift that is particularly consequential in the PLL, where the tissue is inherently poorly vascularized.^31^ As the PLL degenerates, ECM remodeling and structural deterioration further restrict local oxygen diffusion, establishing a sustained hypoxic microenvironment.^32,33^ Under these conditions, HIF-1α drives expression of genes governing angiogenesis (VEGF), glycolytic reprogramming, and cell survival. Critically, HIF-1α also upregulates *Sox9* and *Col2a1*, pushing local cells toward a chondrogenic phenotype. Through cross-talk with osteogenic signaling pathways and reinforcement of pro-inflammatory polarization, these events collectively shape a microenvironment primed for ECM remodeling and pathological ossification.^33–36^ This inflammatory program is further amplified by Toll-like receptor-mediated sensing of damage-associated molecular patterns released in the degenerating PLL, which activates downstream nuclear factor kappa-light-chain-enhancer of activated B cells (NF-κB) and mitogen-activated protein kinase (MAPK) cascades to sustain pro-inflammatory gene expression.^37^ The resulting pro-inflammatory macrophages upregulate surface markers including cluster of differentiation (CD)80, CD86, and MHC class II molecules, and release reactive oxygen species (ROS), reactive nitrogen species, and a broad array of inflammatory mediators, including TNF-α, IL-1β, IL-6, IL-12, and IL-23, that collectively perpetuate local tissue inflammation.^38,39^

Anti-inflammatory macrophages occupy the opposite end of this spectrum, playing a central role in tissue repair and resolution of inflammation. Among their most disease-relevant functions in the OPLL context is their capacity to secrete osteogenic factors that stimulate the differentiation of osteoprogenitor cells and MSCs toward a bone-forming lineage, directly contributing to pathological matrix synthesis.^40^ Underpinning this reparative profile is a distinct metabolic program, as anti-inflammatory macrophages rely on oxidative phosphorylation and fatty acid oxidation driven in part by peroxisome proliferator-activated receptor gamma (PPARγ) signaling that intersects with the lipid metabolism disturbances characteristic of the systemic metabolic dysregulation seen in OPLL.^41,42^ This anti-inflammatory state is further consolidated through Janus kinase (JAK)/signal transducer and activator of transcription 6 (STAT6) signaling, which reinforces the expression of tissue repair-associated and anti-inflammatory genes.^43–45^ A defining feature of anti-inflammatory macrophages is their capacity to promote angiogenesis and vascular remodeling, secreting factors that support endothelial growth and even acquiring endothelial-like traits through transcription factors such as prospero homeobox protein-1 and chicken ovalbumin upstream promoter-transcription factor II.^46^ In particular, VEGF produced by anti-inflammatory macrophages drives metabolic reprogramming and angiogenesis, creating a local hypoxic niche that further amplifies BMP signaling and facilitates endochondral ossification-like processes.^47^ BMPs, conserved members of the TGF-β superfamily, are central to this ossification cascade and well-established drivers of pathological bone formation in heterotopic ossification.^48,49^

Anti-inflammatory macrophages also possess the capacity to undergo direct lineage trans-differentiation under defined microenvironmental conditions, giving rise to myofibroblasts through a process termed macrophage-to-myofibroblast transition.^50^ During this process, anti-inflammatory macrophages progressively acquire the phenotypic and functional characteristics of myofibroblasts, with high expression of α-smooth muscle actin, fibronectin, and collagen, while simultaneously losing their original phagocytic immune function.^51^ The TGF-β/Smad signaling pathway serves as the central regulatory axis of this transition, with Notch, Wnt/β-catenin, and ECM mechanical stress acting as synergistic factors. This has been clearly demonstrated in pulmonary fibrosis, where sustained TGF-β1 stimulation drives alveolar macrophages to transdifferentiate into α-smooth muscle actin-positive myofibroblasts, directly contributing to ECM deposition and progressive fibrotic remodeling.^52^ Moreover, anti-inflammatory macrophages serve as a major source of profibrotic mediators, particularly TGF-β1 and platelet-derived growth factor (PDGF), that drive resident fibroblasts and circulating progenitor cells to differentiate into myofibroblasts. This creates a self-reinforcing cycle, as myofibroblast activation accelerates fibrosis, which progressively alters the local mechanical microenvironment, and the resulting structural changes in turn generate conditions that favor further ossification and tissue remodeling.^53^ The matrix stiffening that accompanies fibrotic remodeling may synergize with the mechanical stress inherent to the spinal column, further activating ligament-resident macrophages in OPLL. These mechanical signals are transduced through integrin/focal adhesion kinase-mediated focal adhesion complexes, triggering Ras homolog family member A/Rho-associated coiled-coil containing protein kinase activation and cytoskeletal reorganization, while mechanosensitive ion channels such as piezo type mechanosensitive ion channel component 1 (Piezo1) mediate calcium influx accompanied by ROS release.^54,55^ These upstream events converge on two core signaling axes. Matrix stiffening promotes inflammatory macrophage polarization while suppressing anti-inflammatory responses through the Piezo1-Yes-associated protein (YAP) axis, whereas mechanical stretch drives the upregulation of pro-inflammatory mediators, including TNF-α, IL-1β, and IL-6, through the Ras homolog family member A/Rho-associated coiled-coil containing protein kinase activation/NF-κB axis.^56,57^ These two pathways may engage in reciprocal cross-talk, as cytoskeletal tension and YAP/transcriptional coactivator with PDZ-binding motif (TAZ) activation can enhance NF-κB transcriptional activity, while inflammatory signals may in turn remodel the ECM and stabilize YAP/TAZ function. Layered on top of this is HIF-1α-driven metabolic reprogramming under the hypoxic conditions of the degenerating PLL. Together, these interactions sustain a chronic pro-inflammatory microenvironment. It should be noted, however, that whether this mechanosensing axis operates directly within PLL-resident macrophages remains to be experimentally validated. These findings collectively illustrate that, despite their extensive tissue-specific heterogeneity, macrophages can drive disease progression through multiple, context-dependent mechanisms, including inflammation, tissue remodeling and fibrosis, as well as angiogenesis and vascular formation, all of which are implicated in OPLL pathogenesis.

### Metabolic dysregulation and macrophage phenotypes in tissues

Metabolic dysregulation has emerged as a major pathogenic driver in spinal ligament ossification,^2,6,7,12–14^ and growing evidence indicates that it critically shapes the macrophage phenotypes contributing to the inflammatory milieu of OPLL. Macrophage metabolism is far more than a housekeeping function, as glucose and lipid metabolic networks not only supply energy and biosynthetic precursors but also actively determine macrophage phenotype by shaping the polarization process.^58,59^ Lipid droplets (LDs) have emerged as dynamic regulators of macrophage inflammatory signaling rather than inert lipid stores. Liberation of arachidonic acid from LDs fuels the synthesis of pro-inflammatory eicosanoids, including prostaglandins and leukotrienes.^60,61^ The enzymes governing LD turnover exert opposing effects on macrophage behavior, with loss of diacylglycerol O-acyltransferase 1 exacerbating inflammatory activation while adipose triglyceride lipase deficiency suppresses inflammation and tilts the balance toward anti-inflammatory polarization.^60^ These findings underscore the importance of LD homeostasis in shaping macrophage fate and inflammatory output, and its vulnerability to disruption under the systemic metabolic conditions closely associated with OPLL, including obesity, dyslipidemia, insulin resistance, and diabetes.

In chronic metabolic-inflammatory diseases such as atherosclerosis, pro-inflammatory macrophages upregulate scavenger receptors, including CD36 and scavenger receptor class A, facilitating the uptake of oxidized low-density lipoprotein, which leads to foam cell formation, cholesterol accumulation, and endoplasmic reticulum stress.^62^ This metabolic burden activates the unfolded protein response and key inflammasome pathways, including NOD-like receptor pyrin domain containing 3 and absent in melanoma 2, driving IL-1β production and sustaining a state of chronic local inflammation.^63^ Central to this process is the cholesterol-sensing transcription factor sterol regulatory element-binding protein-2, which is activated under cholesterol-depleted conditions to restore cholesterol biosynthesis. Sterol regulatory element-binding protein-2 directly engages nod-like receptor pyrin domain containing 3, boosting inflammasome activation and locking cells into a self-amplifying inflammatory loop.^64^ A particularly important downstream product of this pathway is 25-hydroxycholesterol, a sterol intermediate with dual and context-dependent effects. Under normal conditions, it acts as a feedback brake on cholesterol synthesis by inhibiting sterol regulatory element-binding protein processing, but when cholesterol overload and mitochondrial stress converge, it promotes mitochondrial DNA leakage and absent in melanoma 2 inflammasome activation, further amplifying the inflammatory response.^64^ This sustained inflammatory state has direct implications for ossification, as macrophage-driven inflammation stimulates TGF-β1 secretion, which in turn promotes BMP expression in MSCs, a macrophage-dependent mechanism that drives osteogenic differentiation and ultimately contributes to heterotopic ossification, a process with clear mechanistic parallels to the pathological ligament ossification seen in OPLL.^65^ Supporting this, clinical studies consistently associate visceral adiposity, obesity-related metabolic abnormalities, and dyslipidemia with increased OPLL severity and diffuse spinal involvement,^6,7,12^ and OPLL patients carry approximately a twofold higher risk of dyslipidemia compared with healthy controls.^66^

This metabolic-immune axis intersects directly with OPLL genetic susceptibility. *RSPO2*, one of the most robust susceptibility genes identified in OPLL, functions as a potent amplifier of canonical Wnt/β-catenin signaling and thereby promotes endochondral ossification, the central pathological process underlying ligamentous ossification.^3–5^ Macrophages substantially shape the RSPO2-Wnt axis by providing upstream signals that elevate basal Wnt pathway activity. Through their secretion of Wnt ligands such as WNT3A and WNT5A, together with BMP-2, TGF-β1, and pro-inflammatory cytokines including IL-1β and IL-6, macrophages create a pro-osteogenic microenvironment in which RSPO2-mediated amplification of Wnt/β-catenin signaling becomes substantially more pronounced. Another instructive example is IL-6, an adipose-derived cytokine elevated in metabolic syndrome that contributes directly to pathological ossification by promoting osteogenic differentiation of PLL-derived cells, enhancing ALP activity, and upregulating osteogenic transcription factors including *RUNX2* and *SOX9*.^4,67–70^ Together, these findings indicate that systemic metabolic dysfunction skews macrophage polarization toward a pro-inflammatory, lipid-loaded state within spinal ligament tissues, sustaining chronic LGI and creating a microenvironment permissive for aberrant ossification.

### Phagocytosis and defective efferocytosis

As professional phagocytes, macrophages recognize and clear pathogens, foreign particles, necrotic tissue, senescent and apoptotic cells, and cellular debris through a broad repertoire of surface and intracellular receptors that detect danger signals, internalize targets, and degrade engulfed material within phagolysosomes. Beyond pathogen removal, macrophages process phagocytosed antigens for presentation to helper T cells, initiating adaptive immune responses.^71^ The nature of the engulfed cargo further shapes immune outcomes, as phagocytosis can stimulate cytokine and chemokine release that recruits additional immune cells and amplifies local inflammation.^72^ Efferocytosis, the selective recognition and engulfment of apoptotic cells, is mechanistically distinct and immunologically silent, actively preventing inflammation and promoting its resolution.^46^ Macrophages identify apoptotic cells displaying “eat-me” signals and execute engulfment and degradation; impairment of either step can drive excessive cytokine production and disrupt resolution. Failed efferocytosis allows uncleared apoptotic cells to undergo secondary necrosis, releasing DNA, RNA, and other intracellular components that act as endogenous danger signals and sustain chronic inflammation. Notably, advanced glycation end products, which accumulate in aging-associated inflammatory conditions, impair efferocytosis by suppressing key receptors such as MER tyrosine kinase (MerTK) and CD36 while inducing “don’t eat me” signals such as CD47 on apoptotic cells,^46^ further compromising clearance and sustaining a pro-inflammatory tissue environment. Building on this, accumulating evidence links impaired efferocytosis to fibrotic progression across multiple organs, including the lungs in idiopathic pulmonary fibrosis and the liver in chronic liver disease.^73,74^ These fibrotic changes alter local tissue stiffness, vascularization, and cellular composition, collectively creating a permissive niche for aberrant tissue differentiation and ectopic mineralization. Proper efferocytosis is therefore indispensable for maintaining immune homeostasis, and its failure drives a cascade of inflammatory, fibrotic, and autoimmune changes that together generate a microenvironment conducive to pathological ossification. Although direct evidence in OPLL remains limited, these mechanistic parallels strongly suggest that defective efferocytosis may contribute to disease progression by sustaining chronic LGI and fibrotic remodeling within spinal ligament tissues. Elucidating the role of macrophage efferocytosis in OPLL may therefore open a meaningful window into disease mechanisms and point toward novel therapeutic strategies aimed at restoring immune resolution and tissue homeostasis.

### Secretome of macrophages

**Fig. 2:**
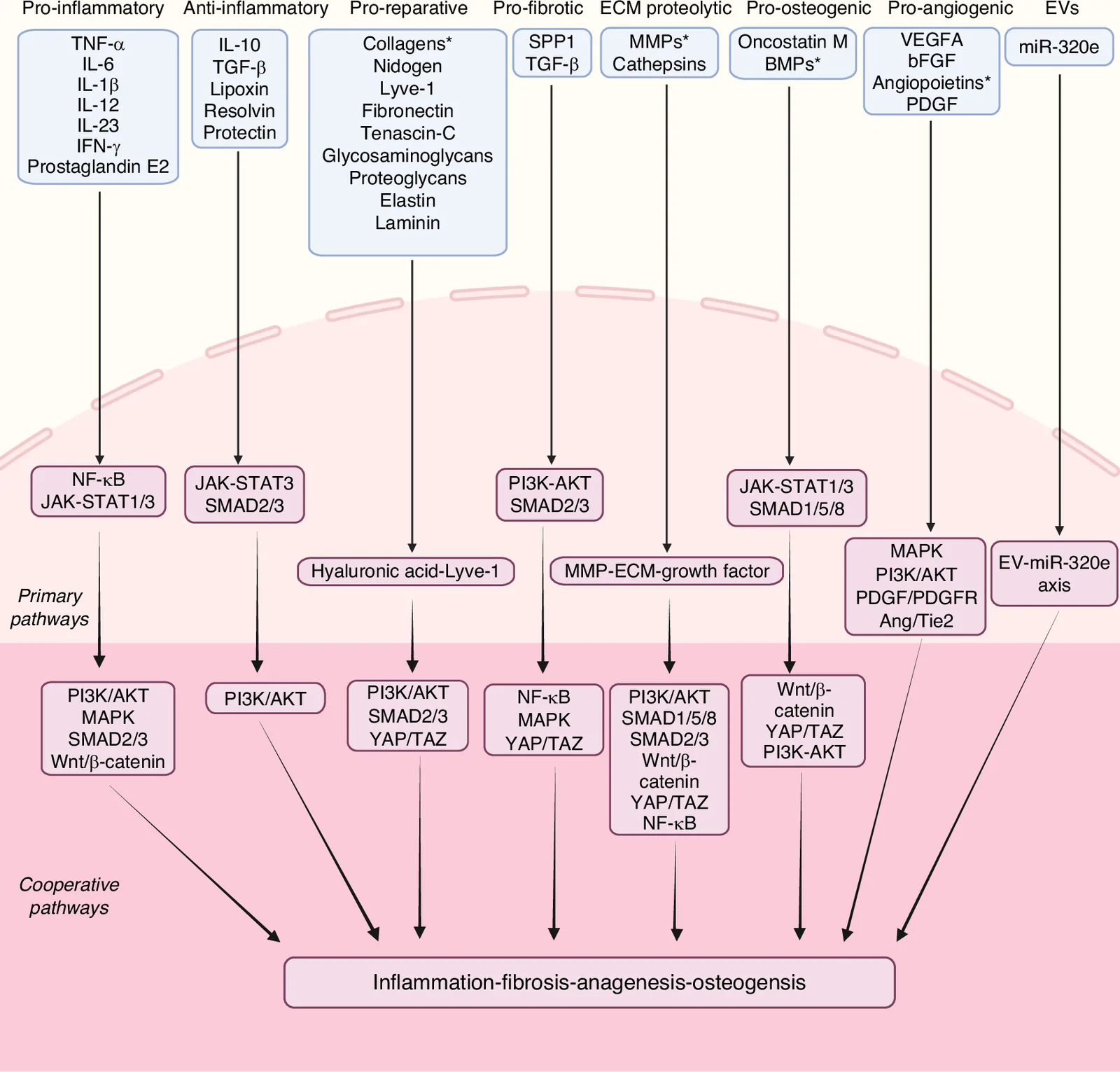
Macrophage-centered signaling network integrating inflammation, fibrosis, angiogenesis, and osteogenesis. Activated macrophages regulate the tissue microenvironment through the release of diverse functional mediators, including pro-inflammatory factors, anti-inflammatory mediators, pro-osteogenic factors, profibrotic factors, ECM-degrading enzymes, reparative matrix components, and pro-angiogenic factors, as well as by delivering pro-osteogenic microRNAs (miRNAs) via extracellular vesicles (EVs). Through extensive cross-talk among multiple signaling pathways, these macrophage-derived signals collectively drive the continuum of inflammatory responses, extracellular matrix remodeling, fibrotic activation, neovascularization, and osteogenic differentiation, thereby contributing to the development of pathological ossification. SPP1 secreted phosphoprotein 1, bFGF basic fibroblast growth factor, Ang angiopoietin, Tie2 tyrosine kinase with immunoglobulin and EGF homology domains 2

Upon activation and polarization, macrophages actively remodel their local microenvironment through diverse secretory pathways, including classical endoplasmic reticulum-Golgi-mediated secretion, extracellular vesicle (EV) release, and the regulated leakage of intracellular metabolites during programmed cell death.^75^ These secretory outputs, referred to as the macrophage secretome, coordinate inflammatory signaling, tissue remodeling, angiogenesis, and osteogenic differentiation (Fig. 2), all of which are central to the pathogenesis of heterotopic ossification and OPLL.

### Pro-inflammatory macrophage-derived mediators

Pro-inflammatory macrophages orchestrate tissue damage by releasing cytokines, including TNF-α, IL-1β, IL-6, Oncostatin M, IL-12, and IL-23, each of which carries distinct downstream consequences that collectively amplify and sustain the inflammatory response.^76^ TNF-α sits at the apex of this cytokine network, functioning as an endogenous pyrogen and master regulator of apoptosis, immune activation, and antimicrobial defense. Its engagement with TNFR1 initiates a death-signaling cascade that can either activate caspase-8 to trigger apoptosis or cleave gasdermin D to drive pyroptosis, two distinct cell death outcomes with important implications for local tissue destruction.^77–79^ IL-6, rapidly induced by tissue injury and immune activation, signals through membrane-bound or soluble IL-6 receptors in complex with gp130, activating JAK/STAT3, MAPK, and phosphoinositide 3-kinase (PI3K)/protein kinase B (AKT) signaling pathways. When STAT3 activation becomes sustained rather than transient, it reinforces inflammatory gene expression and drives the transition from acute to chronic inflammatory pathology.^80^ Oncostatin M, another IL-6 family member secreted by activated macrophages, operates through the same gp130-containing receptor complexes and modulates inflammation, tissue repair, and bone metabolism via STAT1/STAT3 activation, making it a potentially relevant mediator in the OPLL context. Beyond cytokines, pro-inflammatory macrophages reshape the tissue architecture through protease secretion.^81,82^ Matrix metalloproteinase (MMP)-9 is particularly prominent in this regard, degrading a broad spectrum of ECM components while simultaneously modulating inflammation through chemokine activation, neutrophil regulation, and release of latent TGF-β1. Critically, MMP-9 expression is governed by Wnt/β-catenin signaling, directly linking inflammatory macrophage activity to the osteogenic and fibrotic pathways that drive heterotopic ossification.^83,84^

### Anti-inflammatory macrophage-derived mediators

Anti-inflammatory macrophages counter tissue damage through a broad secretory program oriented toward resolution and repair. They release specialized pro-resolving lipid mediators, including lipoxins, resolvins, and protectins, that suppress pro-inflammatory signaling and promote the clearance of apoptotic cells and debris.^85^ Alongside these, they secrete osteogenic factors such as BMP-2 and pro-angiogenic mediators including VEGFA, basic fibroblast growth factor, and angiopoietins-1 and -2, which collectively drive endothelial proliferation, migration, and differentiation.^86^ They also deposit structural ECM components, type I, III, and IV collagens, fibronectin, tenascin-C, and glycosaminoglycans, including hyaluronic acid, heparan sulfate, and chondroitin sulfate, providing the scaffold and microenvironmental support necessary for tissue regeneration.^87^

IL-10, a key anti-inflammatory cytokine derived from anti-inflammatory macrophages, works subtly by activating STAT3 to induce transcriptional repressors that silence pro-inflammatory genes, including TNF, IL-6, IL-1β, and IL-12B, while further limiting interferon regulatory factor-dependent transcription by reducing chromatin accessibility at inflammatory loci.^88^ TGF-β, another major product of anti-inflammatory macrophages, operates at the intersection of immune regulation, fibrosis, and tissue remodeling. Through canonical small mothers against decapentaplegic 2/3 (SMAD2/3)-SMAD4 signaling, it drives expression of α-smooth muscle actin, collagen type I, and tissue inhibitors of metalloproteinases, promoting fibroblast-to-myofibroblast transition and ECM deposition, processes that are directly relevant to the fibrotic remodeling of the PLL in OPLL.^89^ BMP-2, a major macrophage-derived osteoinductive factor warranting particular attention in the OPLL context, signals through both canonical and non-canonical SMAD pathways to activate the master osteogenic transcription factor RUNX2 and engages in cross-talk with Wnt/β-catenin, Notch, and YAP/TAZ mechanotransduction pathways to synergistically drive osteogenesis. This signal is further amplified through the discoidin domain receptor 2-YAP/TAZ axis, which enhances the transcriptional activity of receptor-regulated SMAD complexes.^48^ The pathological relevance of this pathway is underscored by the identification of a pro-ossific F4/80⁺CSF1R^−^ macrophage subset in a murine tibialis anterior injury model that promotes heterotopic ossification formation through BMP signaling. Critically, BMP inhibition suppresses heterotopic ossification development by blocking the BMP-endothelial-to-mesenchymal transition-heterotopic ossification axis.^49^ Together, these findings position macrophage-derived BMP-2 as a potentially central link between the anti-inflammatory macrophage secretome and pathological ligament ossification. VEGFA is a central regulator of vascular development and neovascularization, acting primarily through VEGFR2 to activate downstream MAPK (Ras/Raf/MEK/ERK) and PI3K/Akt cascades that drive endothelial cell proliferation, migration, and survival.^86^ In the OPLL microenvironment specifically, excessive VEGFA-driven neovascularization creates a hypoxic, nutrient-rich niche that amplifies BMP signaling and facilitates the endochondral ossification-like processes.

### EVs and metabolite-mediated signaling

In addition to soluble mediators, macrophages release EVs, including exosomes and microvesicles, which serve as potent vehicles for intercellular communication. EVs carry proteins, lipids, metabolites, mRNAs, and miRNAs and influence immune responses, tissue repair, fibrosis, and metabolic regulation in both paracrine and systemic contexts.^90^ EV cargo composition depends on macrophage activation state, as EVs derived from inflammatory macrophages can exacerbate tissue injury, inducing ferroptosis or pyroptosis in target cells and promoting pathological remodeling, including heterotopic ossification.^91,92^ Likewise, necroptotic macrophage-derived EVs drive osteogenic differentiation and heterotopic ossification in vivo.^93^ In contrast, EVs from anti-inflammatory macrophages generally exhibit anabolic effects, suppressing excessive inflammation and protecting the spinal cord from secondary injury, though they can also promote the trans-differentiation of adipocytes into myofibroblasts, contributing to epidural fibrosis.^93,94^ Of particular relevance to OPLL, small EVs enriched in miR-320e are found abundantly in OPLL tissues, where they promote osteoblastic differentiation of ligament cells and MSCs while simultaneously suppressing osteoclastic differentiation of monocytes, creating a net pro-ossification shift.^95^

Furthermore, beyond classical exocytosis and EV-mediated release of macrophages, emerging evidence suggests that metabolites released during their programmed cell death act as important signaling molecules in surrounding tissues, modulating inflammation, influencing the polarization of immune cells, and promoting tissue remodeling.^96^ For instance, in pyroptotic macrophages, membrane pores formed by cleaved gasdermin D enable the selective release of intracellular metabolites, including 11,12-epoxyeicosatrienoic acid, lactate, and succinate, which activate nearby stem and epithelial cells to initiate regenerative responses.^96^ This mechanism has been particularly well characterized in barrier tissues such as the intestinal epithelium, where these pyroptotic metabolites enhance fibroblast growth factor signaling to drive tissue repair.^96^ Whether analogous metabolite release from apoptotic or pyroptotic macrophages influences osteogenic and fibroblastic differentiation in the spinal ligament, and whether this contributes to or protects against pathological ossification, remains an open and important question for future investigation.

### Macrophages in tissue remodeling

Macrophages are central regulators of tissue remodeling, integrating inflammatory, fibrotic, mechanical, and osteogenic signals to shape the local microenvironment during injury repair and disease progression. By dynamically shifting their activation state and functional phenotype, they coordinate inflammation resolution, ECM turnover, angiogenesis, and the lineage commitment of resident progenitor cells. When this regulatory balance breaks down, as in heterotopic ossification and OPLL, the result is persistent inflammation, aberrant fibrosis, and ultimately ectopic bone formation.

### Inflammation–fibrosis coupling

Inflammation is a protective response that, under physiological conditions, serves as a vital defense mechanism enabling the host to combat infection and tissue injury.^97^ In its early stages, damaged tissues and resident immune cells release bioactive mediators that trigger macrophage activation and polarization. Pro-inflammatory macrophages accumulate at the injury site, secreting cytokines that amplify local inflammation and recruit additional immune cells to contain the threat.^98^ When inflammation becomes excessive or prolonged, macrophage-driven cytokine release sustains fibroblast activation and ECM deposition, tipping the balance toward fibrosis.^99^ This inflammation–fibrosis axis is a defining feature of inflammation-associated heterotopic ossification, where immune cell infiltration in response to tissue injury, neurological insult, or inflammatory stimuli creates a permissive microenvironment for angiogenesis, hypoxia, and osteogenic differentiation.^65^ A key molecular mediator of this transition is secreted phosphoprotein 1, which is highly expressed by macrophages in the early post-injury period. By activating CD44-AKT signaling, secreted phosphoprotein 1 drives myofibroblast differentiation and pathological ECM accumulation, fueling scar formation and fibrotic overgrowth that ultimately prime the tissue for ossification.^100^ A compelling example of inflammation–fibrosis cross-talk is seen in tendon recovery, where the highly fibrogenic tendon microenvironment makes macrophage-mediated fibroblast activation a major obstacle to healing following tissue injury.^101^

Supporting this, clinical observations indicate that nearly 70% of patients with OPLL exhibit heterotopic ossification at extra-spinal sites, including the Achilles tendon, plantar fascia, and ankle ligaments.^102^ This high co-prevalence raises the possibility that repetitive micro-tears in tendons and ligaments may initiate a repair process characterized by inflammation-induced fibroblast activation and excessive ECM deposition, which can subsequently progress to heterotopic ossification during the fibrotic remodeling phase. Macrophages contribute to tendon adhesion primarily through TGF-β1 secretion, which recruits stem cell-derived myofibroblasts to promote this process. Importantly, not all macrophages at the injury site are profibrotic, as a recent study identified FOLR2⁺ macrophages as an anti-fibrotic subset, with atypical chemokine receptor 1 emerging as a key regulator of their trafficking to damaged tissues.^103^ Similar pathological mechanisms are evident in the nervous system, where acute neural injury, driven by ischemia, inflammation, and excitotoxicity, triggers abnormal astrocyte activation that profoundly remodels the local microenvironment and leads to glial scar formation.^104^ This process involves pathological changes, including aberrant ECM deposition, reactive glial proliferation, and excessive secretion of axonal regeneration inhibitors, together establishing formidable physical and biochemical barriers to axon regrowth and neural circuit restoration.^104,105^ These findings collectively reinforce that the inflammation–fibrosis axis is central to classical heterotopic ossification and may also underlie the widespread fibro-ossific tendencies observed in patients with OPLL, with macrophages serving as key orchestrators of this interconnected process.

### Extracellular matrix remodeling

ECM is a complex and dynamic network of macromolecules secreted into the extracellular space, essential for maintaining tissue architecture and function. It is broadly divided into the interstitial matrix, composed largely of type I and III collagens, fibronectin, elastin, and diverse proteoglycans that provide mechanical support and elasticity, and the basement membrane, a dense layer enriched in type IV collagen, laminin, and nidogen that supports epithelial and endothelial cells while regulating cell signaling and barrier integrity.^106^ Far from a passive scaffold, the ECM is now recognized as an active regulator of immune cell behavior, as its molecular composition and mechanical properties shape immune cell adhesion, chemotaxis, and tissue localization through interactions with surface receptors, including integrins, selectins, and glycosaminoglycans.^107^ For example, heparan sulfate engages P- and L-selectins on neutrophils to direct their recruitment to inflammatory sites, while hyaluronic acid binds CD44 on T cells to facilitate their retention and accumulation within inflamed tissues.^108^

Macrophages occupy a unique position at the interface of immunity and ECM remodeling. Tissue-resident macrophage subsets expressing Lyve-1 bind high-molecular-weight hyaluronic acid and contribute to vascular stabilization and perivascular macrophage localization following inflammation.^109^ In response to injury, they remodel the ECM in a stage-dependent manner, with pro-inflammatory macrophages secreting MMPs and cathepsins during the early injury phase to degrade damaged matrix and clear necrotic tissue,^82^ while anti-inflammatory macrophages subsequently take over as inflammation resolves, releasing pro-reparative mediators including TGF-β, PDGF, and VEGF that indirectly drive fibroblast activation and collagen deposition.^98^ Crucially, macrophages also sense ECM tension and composition through integrins and CD44, enabling feedback regulation of their own phenotype and functional state, a mechanosensing loop that further modulates local immune responses and tissue mechanical stability.^110^ A particularly informative parallel comes from the tumor microenvironment, where tumor-associated macrophages, typically displaying an anti-inflammatory phenotype, exhibit potent ECM-remodeling capabilities. Tumor-associated macrophages degrade ECM through a dual mechanism involving both intracellular lysosomal pathways and extracellular MMP secretion, effectively breaching matrix barriers to facilitate tumor cell invasion and metastasis.^111,112^ This process is tightly regulated by vimentin, an intermediate filament protein that coordinates actin stress fiber and podosome dynamics, thereby controlling the membrane localization and activity of membrane-type 1 MMP and shaping the overall ECM-degrading capacity of macrophages. In subcutaneous xenograft models, co-injection of A549 lung cancer cells with either wild-type or vimentin-deficient anti-inflammatory macrophages revealed that only wild-type macrophages drove substantial collagen fiber degradation within the tumor, underscoring vimentin as a critical regulator of macrophage ECM-modulating function.^113^ Together, these examples illustrate conserved macrophage-driven ECM remodeling programs that, when dysregulated in the spinal ligament microenvironment, likely contribute to the structural transformation that culminates in pathological ossification in OPLL.

### Pathological ossification and osteogenic differentiation

Osteogenic differentiation, the process by which MSCs commit to the pre-osteoblast lineage, is tightly orchestrated by key transcription factors including RUNX2 and Osterix.^114^ Within the bone microenvironment, multiple resident cell populations coordinate bone remodeling through paracrine signaling, and macrophages have recently emerged as central regulators of this process.^115^ By secreting chemokines, cytokines, and EVs, macrophages contribute to osteoblast recruitment, differentiation, and function, helping to maintain the continuous remodeling that preserves skeletal architecture and biomechanical integrity. Under pathological inflammatory conditions, however, this balance can be disrupted, allowing aberrant activation of osteogenic pathways and ectopic bone formation in non-skeletal tissues such as the spinal ligament.

The directionality of macrophage influence on MSC differentiation is more nuanced than initially appreciated. Anti-inflammatory macrophages exert sustained pro-osteogenic effects during the later stages of repair, secreting osteoinductive factors that activate canonical pathways including TGF-β/Smad and Wnt/β-catenin to drive osteoblast maturation and ECM mineralization.^68^ Pro-inflammatory macrophages are generally regarded as suppressors of MSC osteogenesis due to their high cytokine output, though during the early inflammatory phase, they can transiently promote osteogenesis through the cyclooxygenase-2-prostaglandin E2-Oncostatin M signaling axis, leading to the upregulation of *RUNX2* and *ALPL* expression in MSCs.^116^ Macrophage-derived EVs add another layer to this regulation, as their uptake by MSCs activates osteogenic pathways that enhance differentiation and support bone tissue regeneration.^117,118^ However, the macrophage-MSC interaction is not unidirectional, as growing evidence indicates that MSCs reciprocally regulate macrophage polarization by secreting factors such as prostaglandin E2 and TGF-β, suppressing the pro-inflammatory phenotype and promoting a shift toward the tissue-reparative anti-inflammatory state.^119^ MSC-derived EVs similarly drive the pro-inflammatory to anti-inflammatory transition, likely through miRNA delivery and mitochondrial transfer, while enhancing macrophage phagocytic and immunomodulatory function.^120^ Together, these bidirectional interactions optimize the regenerative microenvironment and place MSCs and macrophages at the center of the axis linking immune regulation to bone repair.

Beyond biochemical signaling, mechanical cues play an equally decisive role in shaping macrophage function and osteogenic responses. Macrophage activity in bone formation is regulated by mechanosensitive pathways, including those mediated by integrins and YAP/TAZ.^121^ YAP/TAZ are nuclear effectors that translate mechanical signals from ECM stiffness, cellular geometry, and cytoskeletal tension into transcriptional output. On stiff ECM substrates or in highly spread cellular configurations, YAP/TAZ translocate to the nucleus and activate target genes such as *CTGF* and *ANKRD1*. In contrast, on soft matrices or within geometrically constrained environments, YAP/TAZ are retained in the cytoplasm and remain inactive. This regulation depends on Rho GTPase activity and actomyosin tension and operates independently of the canonical Hippo/large tumor suppressor kinase pathway.^122^ Through this mechanotransduction axis, macrophages remodel the ECM and modulate the mechanosensory responses of ligament fibroblasts, amplifying the influence of mechanical loading on ossification in diseases such as OPLL.^110^ Macrophages themselves are not just transmitters of mechanical signals but also responders, as YAP/TAZ signaling within macrophages directly regulates their polarization programs and secretory profiles, reinforcing their central position at the immune-mechanical-osteogenic interface.^56^ A particularly intriguing recent observation is that increased ECM stiffness does not simply amplify repair responses; paradoxically, it suppresses the expression of pro-repair genes such as *Fizz1* and *Rnase2a*, thereby inhibiting the macrophage tissue repair program. This regulation depends on cytoskeletal remodeling and chromatin accessibility changes, forming a negative feedback mechanism that may serve to prevent excessive ECM deposition and runaway fibrosis.^123^ In the OPLL setting, however, where this feedback may itself become dysregulated, the consequences for macrophage-driven matrix remodeling and ossification warrant further investigation.

### Osteoinflammation paradox

The relationship between inflammation and bone metabolism is not strictly unidirectional. During the early phase of acute inflammation, macrophage-derived pro-inflammatory cytokines, including TNF-α, IL-1β, and IL-6, can transiently promote osteogenic differentiation of mesenchymal progenitors through BMP signaling activation, contributing to bone formation.^124^ As inflammation persists and transitions into a chronic state, however, these same mediators shift their function toward sustaining osteoclastogenesis through persistent activation of the receptor activator of nuclear factor κB ligand (RANKL) axis, ultimately driving net bone resorption.^125^ This temporal duality of cytokine action gives rise to a fundamental paradox within the classical osteoimmunological framework. While inflammatory conditions are conventionally associated with osteoclast-driven bone loss, as exemplified by rheumatoid arthritis (RA) and related erosive arthropathies, patients with OPLL paradoxically develop pathological ectopic ossification within an apparently similar inflammatory milieu. Among inflammatory bone disorders, spondyloarthritis (SpA) offers the most relevant comparison to OPLL, sharing closer pathomechanistic parallels than RA. SpA encompasses a heterogeneous group of chronic inflammatory disorders unified by enthesitis as their cardinal feature, predominantly affecting the spine, sacroiliac joints, and the fibrocartilaginous insertion sites of tendons and ligaments. Genetic predisposition forms the primary etiological substrate, with human leukocyte antigen B27 (HLA-B27) representing the central susceptibility locus.^126^ In early-stage SpA, entheseal inflammation and bone erosion predominate, driven by TNF-α and IL-17A-mediated activation of the RANKL axis and subsequent osteoclastogenesis.^127^ As the disease progresses, mechanically induced microdamage at entheseal sites engages in synergistic cross-talk with IL-23/IL-17 axis-driven immune activation, sustaining chronic inflammation and eventually triggering aberrant osteoproliferative programs as inflammation begins to resolve. In contrast, RA operates within a sustained high-grade inflammatory microenvironment with a markedly different outcome. Its pathological core resides in the chronically inflamed synovium, where infiltrating T cells, B cells, and macrophages perpetually elaborate TNF-α, IL-1β, and IL-6, driving an elevated RANKL/osteoprotegerin (OPG) ratio that sustains osteoclast activation and ultimately produces periarticular cortical bone erosion.^128^ Biomechanical loading drives chemokine release from mesenchymal stromal cells, facilitating classical monocyte recruitment and osteoclast differentiation, and thereby determining the site-specific distribution of inflammatory tissue damage.^129^ Despite sharing broadly overlapping inflammatory cytokine profiles, with sustained TNF-α and IL-6 overexpression as a common denominator, SpA and RA diverge fundamentally in their skeletal consequences.^130,131^ RA manifests as pure pathological osteolysis, whereas SpA is characterized by concurrent bone erosion and aberrant new bone formation that ultimately progresses toward ankylosis. This divergence likely reflects a fundamental difference in the mechanical environment between SpA entheseal sites and RA synovial joints. Entheses are subjected to mixed tensile-compressive loading transmitted through tendons and ligaments, and this distinct mechanobiological context cooperates with inflammatory mediators to redirect bone metabolic programs from a resorptive toward an osteogenic trajectory.^130–134^ Accumulating evidence demonstrates that cyclic tensile strain harbors intrinsic osteoinductive potential, capable of activating BMP, Wnt/β-catenin, and MAPK signaling cascades while upregulating osteogenic markers including *RUNX2* and *BMP2*.^134,135^ In preclinical SpA models, hindlimb unloading substantially attenuates Achilles entheseal inflammation, whereas reambulation produces marked exacerbation of both enthesitis and new bone formation in proportion to inflammatory severity, a process mediated principally through stromal rather than adaptive immune cells, with extracellular signal-regulated kinase 1/2 (Erk1/2) signaling serving as a critical mechanotransduction node.^133^ The mechanosensitive ion channel Piezo1 is aberrantly upregulated in ligamentous and entheseal tissues of patients with ankylosing spondylitis (AS), and its activation drives pathological new bone formation through calcium/calmodulin-dependent protein kinase II (CaMKII) signaling. Critically, AS-associated inflammatory cytokines further upregulate Piezo1 expression, amplifying mechanosensitivity and reinforcing downstream osteogenic programs, establishing a self-reinforcing mechanoinflammatory circuit that underlies entheseal ossification in SpA-AS.^136^

OPLL shares with SpA the fundamental pathological substrate of a chronic inflammatory background superimposed upon sustained mechanical loading, but with a critical mechanical distinction. Unlike the mixed compressive-tensile environment of SpA entheseal sites, the PLL, a longitudinal ligamentous structure anchoring the posterior vertebral bodies, experiences predominantly cyclic tensile strain during repetitive cervical flexion.^137^ This mechanobiological distinction may underlie the divergent ossification program of OPLL relative to SpA enthesopathy. The tensile mechanical milieu confers upon the PLL microenvironment an intrinsic osteogenic drive, and sustained mechanical loading further activates the YAP/TAZ signaling axis, reinforcing osteogenic commitment of resident mesenchymal progenitors.^138^ Within the PLL microenvironment of OPLL, chronic inflammation establishes the permissive cellular activation background, while tensile strain-driven BMP/Wnt/MAPK osteogenic signaling and YAP/TAZ-mediated mechanotransduction cooperatively constitute the core switching mechanism that redirects inflammatory signals toward osteogenic programs. In a manner paralleling the genetic architecture of SpA susceptibility, OPLL-specific susceptibility loci, including *RSPO2* and *SNX17*, may confer heightened cellular sensitivity to BMP/Wnt osteogenic signaling within PLL-resident cells, enabling ossification programs to be initiated at comparatively lower signal thresholds.^3,4^ This genetic predisposition provides a partial mechanistic explanation for why, under comparable inflammatory and mechanical conditions, PLL cells exhibit a preferential propensity toward osteogenic commitment that ultimately drives OPLL pathogenesis.

## Macrophages as targets for therapeutic opportunities

Macrophages represent highly attractive therapeutic targets in OPLL, as their remarkable plasticity and central positioning at the interface of inflammation, fibrosis, angiogenesis, and osteogenesis enable them to orchestrate disease progression. Advances in immunology and bioengineering have identified well-defined signaling pathways, transcriptional regulators, recruitment mechanisms, and effector molecules that govern macrophage activation and function, rendering macrophage-centered therapeutic strategies both feasible and conceptually compelling (Table 1). Targeted modulation of macrophage phenotypes, therefore, offers an opportunity to simultaneously dampen pathological inflammation while disrupting the permissive microenvironment that drives aberrant ossification.

**Table. 1:** Pharmacological strategies modulating macrophage signaling and polarization in ossification disorders

Drug	Mechanism	Therapeutic effect (experimental model)	Clinical identifier/intervention	Trial Phase/Reference
Garcinol	Synergistically blocks the NF-κB signaling pathway and histone H3K9 acetylation	Alleviates joint inflammation and modulates macrophage polarization (CIA mouse model)	CTRI/2019/11/022147; (Garcinol, Curcuminoids, Piperine)	Phase II/^140,156^
Rapamycin	Inhibits mTORC1 activity and restores Akt activity	Restores M2 macrophage polarization capacity and suppresses excessive pro-inflammatory responses in vivo (Myeloid-specific *Tsc1* knockout-induced mTORC1 activation mouse model)	NCT03882437; (RP-A501)	Phase I/^142,157^
Metformin	Activates the AMPK signaling pathway, improves mitochondrial function	Attenuates the pro-inflammatory activation of macrophages and significantly ameliorates acute pulmonary inflammatory responses (BMDMs)	ACTRN12621000710820; (Metformin extended release)	NA/^144,158^
Aspirin/Sodium Salicylate	Inhibition of NF-κB p65 to block *SLC7A11* transcription	Suppression of *SLC7A11* expression to inhibit hepatocellular carcinoma growth (HepG2 and Huh7 cells/HCC xenograft mouse model)	NCT03377543; (Aspirin)	NA/^154,159^
Dexamethasone	Interaction with the glucocorticoid receptor to block NF-κB DNA binding	Reprogramming of dendritic cells to a tolerogenic phenotype to block the progression of arthritis (BMDCs)	NCT03710603; (Daratumumab, Bortezomib, Lenalidomide, Dexamethasone)	Phase III/^160,161^
Sorafenib	Inhibits VEGFR/PDGFR and uncouples LOXL2-mediated vascularization and osteogenesis	Suppression of heterotopic ossification in the posterior longitudinal ligament and alleviation of neurological deficits in OPLL (primary human ligament cells/BMP-induced ossification mouse model/*Enpp1*-deficient spontaneous OPLL mouse model)	NCT00105443; (Sorafenib/Nexavar)	Phase III/^24,162^
Matrine	Inhibits the MAPK signaling pathway	Reduces heterotopic bone formation and decreases expression of osteogenic markers (Burn and Achilles tendon transection-induced traumatic HO mouse model)	ChiCTR-TRC-14004301; (Oxymatrine)	NA/^152,163^
Garetosmab (ActA-mAb)	Blocks Activin A-BMP axis	Reduces osteogenic markers; decreases bone formation area (FOP mouse model)	NCT03188666; (Garetosmab)	Phase II/^164^
JNJ-40346527	Inhibiting CSF1R reduces monocyte recruitment/macrophage infiltration	Inhibiting microglial proliferation and stopping neurodegeneration (P301S transgenic mouse model/ME7 prion disease model)	NCT01597739; (JNJ-40346527)	Phase IIA/^149,165^
Verteporfin	Targeting the YAP/SMAD complex to block TGF-β signaling	AT2 cells to a non-fibrotic phenotype to block the progression of IPF (AT2 cells/C57BL/6 mouse model)	NCT03033225; (Verteporfin PDT)	Phase I/II/^166,167^

These agents are currently approved primarily for malignant indications, and their inclusion here serves as a mechanistic proof-of-concept only; direct clinical application in OPLL would require careful consideration, given the chronic, non-malignant nature of the disease

*Akt* serine/threonine kinase, *AMPK* AMP-activated protein kinase, *AT2* alveolar type II, *BMDMs* bone marrow-derived macrophages, *BMDCs* bone marrow-derived dendritic cells, *BMP* bone morphogenetic protein, *CIA* collagen-induced arthritis, *CSF1R* colony-stimulating factor 1 receptor, *Enpp1* Ectonucleotide pyrophosphatase/phosphodiesterase 1, *FOP* Fibrodysplasia ossificans progressiva, *HCC* hepatocellular carcinoma, *HO* heterotopic ossification, *IPF* idiopathic pulmonary fibrosis, *LOXL2* lysyl oxidase-like 2, *MAPK* mitogen-activated protein kinase, *mTORC1* mechanistic/mammalian target of rapamycin complex 1, *NF-κB* nuclear factor-κB, *OPLL* ossification of the posterior longitudinal ligament, *PDGFR* platelet-derived growth factor receptor, *SLC7A11* Solute carrier family 7 member 11, *SMAD* mothers against decapentaplegic homolog, *TGF-β* transforming growth factor-β, *Tsc1* tuberous sclerosis complex 1, *VEGFR* vascular endothelial growth factor receptor, *YAP* yes-associated protein

### Targeting signaling and transcriptional networks

**Fig. 3:**
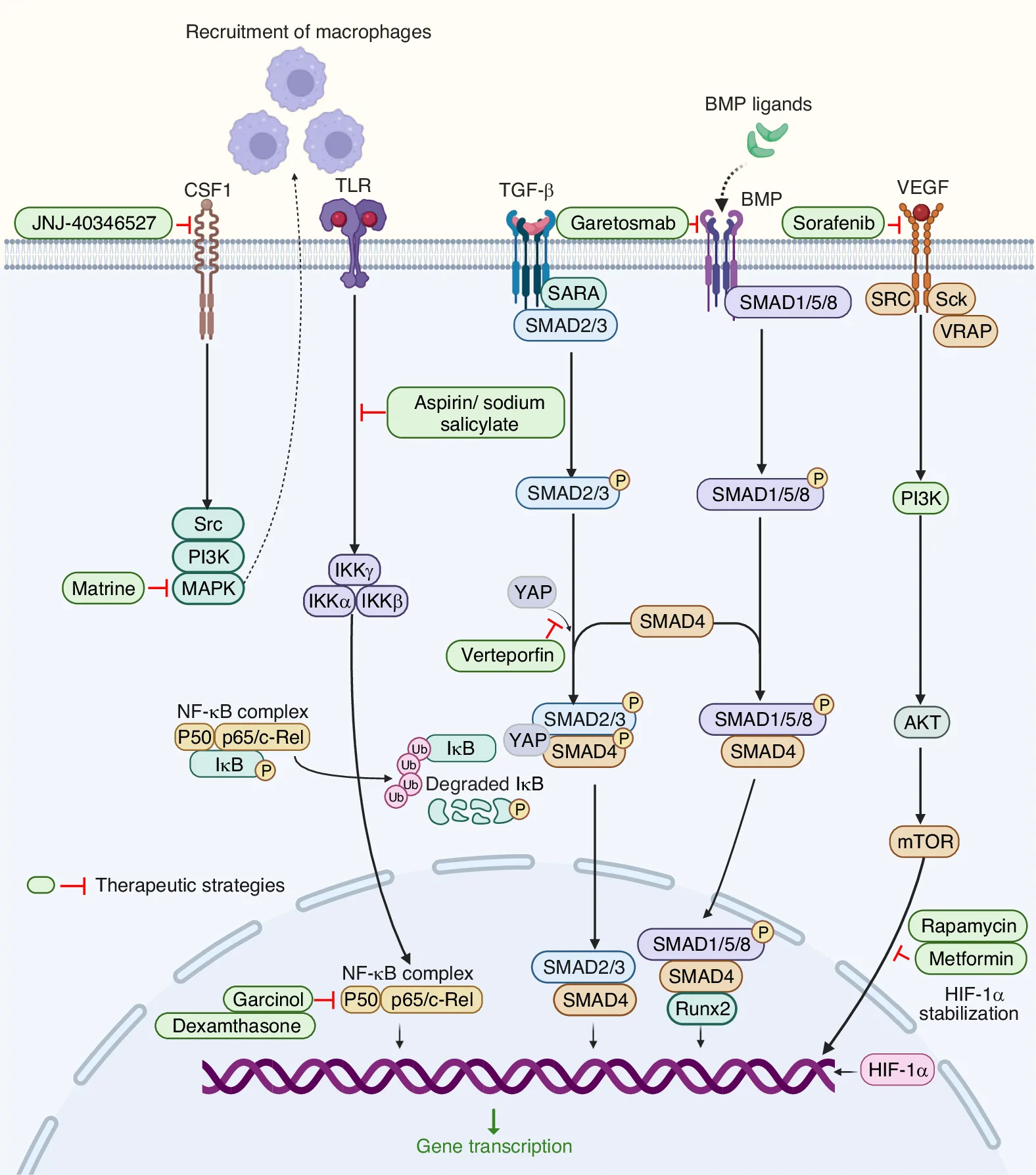
Signaling pathways and therapeutic strategies targeting macrophage polarization and secretome. Multiple signaling pathways and transcription factors, including nuclear factor-κB (NF-κB), mitogen-activated protein kinase (MAPK), mechanistic target of rapamycin (mTOR), transforming growth factor-β/small mothers against decapentaplegic (TGF-β/SMAD), and BMP/SMAD, play pivotal roles in regulating metabolic reprogramming and phenotypic switching of macrophages. Targeting macrophage polarization and their secretome to modulate inflammatory responses may ultimately suppress pathological ossification. Therapeutic strategies include blocking pro-inflammatory macrophage-induced signaling, enhancing the anti-inflammatory effects of macrophages, inhibiting osteogenic differentiation of MSCs, suppressing osteogenic microvascular formation, and targeting macrophage recruitment and effector molecule secretion

Targeting macrophage function through specific signaling pathways, transcription factors, or subpopulations, and via approaches such as relay transfer and cell transplantation, has emerged as a promising therapeutic strategy for promoting tissue repair and regeneration (Fig. 3). Macrophages exhibit remarkable plasticity, capable of dynamically switching between pro-inflammatory and anti-inflammatory phenotypes. This functional heterogeneity enables macrophages to coordinate immune responses and tissue remodeling in concert with the local microenvironment, ultimately leading to either physiological repair or pathological remodeling. Harnessing this plasticity and achieving precise regulation of macrophage polarization has become a key strategy in the treatment of various diseases.^139^ A growing body of research has focused on targeting macrophage polarization to modulate the downstream inflammatory and reparative pathways that shape osteogenesis. The p300/CBP-associated factor (PCAF) provides an instructive example. PCAF acts as an upstream regulator of pro-inflammatory polarization, promoting transcription of pro-inflammatory genes by modulating histone H3K9 acetylation and activating the NF-κB signaling pathway. In primary macrophages, PCAF expression correlates positively with inflammatory mediators including IL-6, TNF-α, and inducible nitric oxide synthase (iNOS).^140^ In collagen-induced arthritis mouse models, pharmacological inhibition of PCAF with garcinol effectively suppresses pro-inflammatory macrophage-associated factors, reduces histone H3K9 acetylation levels in synovial macrophages, alleviates synovial inflammation, and limits tissue damage. As inflammation subsides under this treatment, the local environment becomes enriched in anti-inflammatory macrophages, a shift that is essential for establishing a bone-forming niche.^140^

In addition to epigenetic regulation, metabolic signaling pathways represent another key determinant of macrophage polarization states. The mTOR pathway serves as a central regulatory axis shaping macrophage metabolism and function. In pro-inflammatory macrophages, activation of mTOR complex 1 (mTORC1) promotes glycolytic metabolism and drives a pro-inflammatory phenotype by inducing HIF-1α and glycolytic enzyme expression.^141,142^ When mTORC1 is inhibited by rapamycin or inactivated through *Raptor* deletion, macrophages undergo a phenotypic shift to an anti-inflammatory phenotype, accompanied by restoration of AKT signaling and upregulation of genes such as *Arg1* and *Fizz1*, resulting in an anti-inflammatory phenotype.^142^ AMPK, an evolutionarily conserved energy sensor, acts as a major upstream regulator of mTORC1. Upon activation, AMPK phosphorylates tuberous sclerosis complex 2 to inhibit mTORC1 activity and redirects signaling toward the mTORC2–AKT pathway.^143^ This promotes IL-10 production through the AKT–glycogen synthase kinase 3β-cAMP response element-binding protein axis while suppressing pro-inflammatory cytokines such as TNF-α and IL-6. In parallel, AMPK inhibits fatty acid synthesis and enhances fatty acid oxidation and mitochondrial oxidative metabolism, providing the metabolic foundation that supports anti-inflammatory functions and polarization.^143^ Metformin, a classical activator of AMPK, has been extensively investigated in both animal and cell models. In bone marrow-derived macrophages, metformin enhanced the p-AMPK/AMPK ratio and selectively dampened the acute pro-inflammatory response through an AMPK-dependent mechanism.^144^ Moreover, PPARs play a crucial role in the metabolic regulation of macrophage polarization, with PPARγ acting as a key driver of anti-inflammatory polarization. PPARγ directly binds to PPAR response elements in the promoters of anti-inflammatory signature genes, such as *Arg1*, enhancing their transcription and promoting the expression of anti-inflammatory markers.^145^ PPARγ further reprograms macrophage metabolism by upregulating genes related to fatty acid uptake (*CD36*), fatty acid oxidation (*Acadm*, *Acadl*), and mitochondrial biogenesis, thereby sustaining oxidative metabolism and supporting anti-inflammatory polarization. In addition, PPARγ suppresses lipopolysaccharide-induced IL-6 production, reinforcing the anti-inflammatory phenotype. In contrast, loss of PPARδ does not affect IL-4-induced fatty acid oxidation or anti-inflammatory-associated metabolic reprogramming, underscoring the specificity and dominant role of PPARγ in this process.^145^

NF-κB sits at the center of a broad inflammatory network. Its activation drives sustained production of pro-inflammatory cytokines including TNF-α, IL-1β, and IL-6, and through positive feedback loops with the MAPK and JAK-STAT pathways, it amplifies inflammatory cascades well beyond their initial trigger.^146^ This central position makes NF-κB an attractive therapeutic target. Several mechanistic approaches have been explored. IKK inhibitors such as aspirin and sodium salicylate block IκB phosphorylation and degradation, retaining NF-κB in the cytoplasm. Proteasome inhibitors such as bortezomib prevent IκBα degradation and thereby block NF-κB nuclear translocation. Glucocorticoids such as dexamethasone bind directly to the RelA subunit of NF-κB, blocking its interaction with DNA promoters. Each of these strategies has demonstrated potent anti-inflammatory activity across diverse disease models. Importantly, because NF-κB is so deeply interconnected with the MAPK and JAK-STAT pathways, its inhibition produces synergistic effects across multiple signaling axes rather than acting through a single pathway alone.^146,147^

### Blockade of macrophage recruitment

Beyond intracellular reprogramming, limiting the excessive recruitment of macrophages to inflamed tissues offers another promising therapeutic angle. The C-C motif chemokine ligand 2 (CCL2)/C-C motif chemokine receptor 2 (CCR2) axis and colony-stimulating factor 1 receptor signaling are central to monocyte mobilization, macrophage survival, and tissue infiltration,^148,149^ and pharmacological blockade of either pathway reduces macrophage accumulation, dampens inflammatory cytokine output, and reshapes the local immune microenvironment.^149^ In experimental models, disrupting CCR2/CCL2 signaling significantly reduces infiltration of inflammatory monocytes and tissue macrophages, attenuating local inflammation and helping restore immune balance.^150^ Colony-stimulating factor 1 receptor inhibitors act through a complementary mechanism, impairing macrophage survival and recruitment. Given the documented macrophage enrichment in ossified ligament tissues, targeting macrophage recruitment may represent an upstream intervention point to prevent the establishment of an ossification-permissive niche in OPLL.

### Inhibiting macrophage-derived effector factors

Macrophage-derived effector molecules play a decisive role in shaping the osteo-inflammatory microenvironment. Different macrophage phenotypes exhibit distinct secretory profiles, including chemokines, matrix-degrading enzymes, angiogenic factors, and EVs. These secretomes promote osteogenic differentiation of tendon-derived stem cells, thereby driving pathological bone formation. Anti-inflammatory macrophages play a critical role in pathological ossification as a major cellular source of VEGF and PDGF-BB within the osteo-inflammatory microenvironment, driving angiogenesis through the recruitment of pericytes and mesenchymal progenitors, which together create the vascular and cellular framework that ultimately supports pathological osteogenesis.^151^ Single-cell RNA sequencing of OPLL lesion specimens, supported by clinical sample validation, has revealed marked upregulation of lysyl oxidase-like 2 (*LOXL2*), which activates the HIF-1α pathway to drive secretion of VEGFA and PDGF-BB, closely recapitulating the pro-angiogenic effector profile characteristic of anti-inflammatory macrophage-driven ossification. Sorafenib, a broad-spectrum tyrosine kinase inhibitor, effectively suppresses LOXL2-mediated vascular morphogenesis. In both BMP-induced and *Enpp1*-deficient OPLL animal models, sorafenib significantly inhibited OPLL progression without affecting normal bone mass, suggesting that pharmacological disruption of the macrophage-associated angiogenic axis may offer a viable strategy for limiting pathological ossification in OPLL.^24^

A central limitation must be acknowledged before discussing specific pharmacological strategies. The majority of mechanistic studies in this area have been conducted either in acute traumatic heterotopic ossification models or in the rare genetic disorder fibrodysplasia ossificans progressiva (FOP), which is characterized by gain-of-function mutations in *ACVR1*.^152,153^ OPLL, in contrast, is a chronic, age-related degenerative process that unfolds over years within the distinct biochemical and biomechanical niche of the PLL.^1–3^ The PLL also harbors its own susceptibility factors, including genetic loci such as *RSPO2* and *SNX17*, that may render it particularly prone to ossification independently of acute inflammation.^3,4^ Mechanistic inferences drawn from acute heterotopic ossification or FOP models should therefore be regarded as hypothesis-generating rather than directly validated in OPLL, and future studies using OPLL-specific specimens and models will be essential to establish causal relationships. With this caveat in mind, the studies discussed below offer useful mechanistic anchors that can guide the rational development of OPLL-targeted therapies. One investigation highlighted the temporal dynamics of macrophage polarization across different stages of heterotopic ossification, extending both the mechanistic understanding and the therapeutic window for intervention.^65^ In a traumatic heterotopic ossification mouse model, the alkaloid matrine was shown to downregulate TGF-β and VEGF expression in anti-inflammatory macrophages and significantly reduce the volume and maturity of ectopic bone.^152^

Targeting macrophage-derived effector molecules has likewise emerged as a promising therapeutic avenue. The Activin A-BMP signaling axis is a central driver of heterotopic ossification in FOP, and blocking either Activin A or BMP ligands can markedly reduce heterotopic ossification formation.^153,154^ Hatsell and colleagues demonstrated in an FOP mouse model that treatment with activin A-neutralizing antibodies or BMP ligand traps such as ACVR1 inhibitors effectively suppressed both spontaneous and trauma-induced heterotopic ossification, reduced osteogenic marker expression, and decreased bone formation area.^153^ Supporting this, Huang and colleagues reported that areas of macrophage infiltration overlapped spatially with BMP-7 expression in heterotopic ossification lesions, and that macrophage depletion significantly reduced both local BMP-7 levels and bone formation, positioning macrophages as upstream amplifiers of BMP signaling.^65^

## Concluding remarks and future perspectives

Accumulating evidence positions OPLL as more than a localized degenerative disorder; it appears to represent a manifestation of systemic immune dysregulation in which chronic inflammation plays a central pathogenic role. Within this inflammatory milieu, macrophages have emerged as pivotal regulators of disease initiation and progression. Pathological ossification is increasingly understood as a terminal outcome of inflammation-driven fibrotic remodeling in non-osseous tissues, marked by aberrant lineage reprogramming of resident cells and progressive deposition of bone-like matrix. Across this continuum, macrophages act as central cellular integrators, orchestrating early inflammatory responses, shaping fibroblast activation and angiogenesis, and ultimately steering pathological osteogenic differentiation through context-dependent polarization states and secretory programs. In doing so, they forge the critical mechanistic links between inflammation, fibrosis, and ectopic ossification.^155^

Despite growing interest in macrophage-centered therapeutic strategies, our understanding of the molecular mechanisms governing macrophage function in OPLL remains incomplete. Future investigations should prioritize defining the temporal and spatial dynamics of macrophage phenotypes across distinct stages of OPLL progression. Particular attention is warranted for the interplay between macrophage immunometabolic reprogramming, especially glucose and lipid metabolism, and canonical osteogenic signaling pathways, including BMP, Wnt/β-catenin, and TGF-β. In parallel, the functional contributions of macrophage-derived EVs and their metabolic cargo to fibro-osteogenic remodeling within the ligament microenvironment warrant systematic investigation. Equally important is elucidating how macrophage metabolic states influence their capacity to regulate fibrosis and osteogenesis within the unique biomechanical and biochemical niche of the PLL. Dissecting the reciprocal interactions among immune activation, metabolic rewiring, and bone formation may reveal upstream regulatory nodes that can be therapeutically exploited to disrupt disease progression. Targeting macrophage polarization, recruitment, or secretory activity, therefore, represents a promising strategy to halt or slow pathological ossification in OPLL, and continued integration of immunology, metabolism, and targeted pharmacological approaches is expected to accelerate the development of disease-modifying therapies.

In summary, macrophages occupy a central position at the intersection of inflammation, fibrosis, and pathological ossification. Advancing our understanding of macrophage immunometabolic regulation and intercellular communication within the ligament niche may ultimately enable the development of targeted interventions capable of preventing or even reversing OPLL progression.
